# Law as a Tool for Preventing Chronic Diseases: Expanding the Spectrum of Effective Public Health Strategies: Part 2

**Published:** 2004-03-15

**Authors:** George A. Mensah, Richard A. Goodman, Stephanie Zaza, Anthony D. Moulton, Paula L. Kocher, William H. Dietz, Terry F. Pechacek, James S. Marks

**Affiliations:** Cardiovascular Health Branch, Division of Adult and Community Health, National Center for Chronic Disease Prevention and Health Promotion, Centers for Disease Control and Prevention; Public Health Law Program, Public Health Practice Program Office; Office of the Director, NCCDPHP; Public Health Law Program, Public Health Practice Program Office; Office of General Counsel; Division of Nutrition and Physical Activity, NCCDPHP; Office of Smoking and Health, NCCDPHP; NCCDPHP

## Introduction

In part one of this 2-part series, we reviewed the important roles that laws have played in public health and provided examples of specific laws and their effectiveness in supporting public health interventions (1). We suggested that conceptual legal frameworks for systematically applying law to preventing and controlling chronic diseases have not been fully recognized and we provided the basic elements of a conceptual legal framework. In part 2 of this series, we first provide an overview of U.S. jurisprudence, describe the legal mechanisms, remedies, and tools for applying law to public health, and summarize the jurisdictional levels at which laws, mechanisms, remedies, and tools operate. We then identify the potential contours for legal frameworks of varying complexity and scope by offering examples of legal frameworks in public health practice. This paper also outlines a plan for increasing the capacity within the Centers for Disease Control and Prevention (CDC) for developing legal frameworks and expanding guidance on using legal tools for preventing and controlling chronic diseases. Finally, we describe resources for building or enhancing the capacity to use law as a tool for preventing diseases, injuries, and disabilities at the local level.

## Overview of U.S. jurisprudence and legal methods relevant to public health

The dimensions and elements of a systematic legal framework for preventing chronic diseases and other public health problems can be drawn from examining relevant fields of U.S. jurisprudence, legal theories, and legal methods. These dimensions and elements include the following: 1) basic sources of U.S. law relevant to preventing and controlling public health problems; 2) legal mechanisms, remedies, and tools for applying law to disease prevention and control; and 3) jurisdictional levels at which such laws, mechanisms, and tools might be appropriately applied.

### Basic sources of U.S. law relevant to public health

Basic sources of U.S. law include the federal Constitution and state constitutions, federal and state legislative enactments, formally ratified treaties, administrative law promulgated and enforced by agencies to which legislatures have delegated such authorities, and common law (also frequently referred to as case law) articulated by the federal and state judiciary following appellate review. While the term "general welfare" is mentioned twice in the U.S. Constitution — the supreme law of the land — nowhere is the term "public health" mentioned. This absence may possibly reflect the view at the time the Constitution was established that protection of the public’s health was a state responsibility and not a duty to be assigned to the national government (2). In addition, through police powers — reserved to the states by the Tenth Amendment — states retained responsibility for public health (3,4). Despite the absence of the term in the Constitution, several provisions do confer some public health powers on the federal government as well as affect the exercise of police power by the states. For example, one provision (Article I, Section 8) confers on Congress the powers to tax, appropriate monies, and provide for the general welfare of the United States (3). These authorities have enabled Congress to establish agencies with responsibilities in public health within the executive branch, as well as to allocate monies earmarked for public health activities to the states.

In contrast to the U.S. Constitution’s grant of limited, enumerated powers to the federal government, individual state constitutions exist as limits on the sovereign powers of states. While state constitutions vary in their references to public health, many provide for their legislatures to establish state — and sometimes county or local — boards of health. In addition, states may delegate public health responsibilities to such local authorities.

A combination of federal statutes establishes roles and authorities of federal agencies in disease prevention and control activities. The CDC and the Food and Drug Administration (FDA) are 2 such agencies for which Congress has statutorily conferred explicit public health responsibilities and authorities to address public health problems. The responsibilities and powers of these agencies are reflected in provisions of the Public Health Service Act (PHSA) and the Food, Drug, and Cosmetic Act. Sections of Title III of the PHSA encompass a range of powers and duties for disease control and prevention, including conducting scientific research relating to causes, treatment, control, and prevention of diseases (Section 301), and for federal–state cooperation in disease prevention and control (Section 311). Examples of PHSA provisions more targeted to chronic disease issues are Section 317H, which covers surveillance and juvenile diabetes, Sections 399W-Z, which cover programs to improve the health of children, including, for example, grants to promote childhood nutrition and physical activity, and applied research into childhood obesity, and Sections 1501-1510, which cover breast and cervical cancer screening.

State legislatures, by acting under their broad plenary authorities and by expressing their police powers to protect the health and safety of their populations, enact numerous statutes for disease control and prevention. These statutes create public health agencies at state and local levels, articulate express authorities for such agencies to assure the public’s health through regulatory and non-regulatory actions, and may even delegate such powers to lower-level agencies. While the states’ legal authorities for preventing and controlling many infectious diseases have been comprehensively described (5), such information has not been well-characterized for chronic diseases.

In addition to constitutions and statutes, other important sources of law affecting public health include administrative law and common law. Administrative law is created by administrative agencies through rules, regulations, orders, and procedures designed to promote policy goals enacted by legislation (6). Responsibility for implementing and enforcing such regulations may be delegated by legislatures to public health departments and other regulatory agencies which, through the processes of issuing and enforcing regulations, create a body of administrative law. Administrative law requirements may govern a spectrum of public health actions that range from the designation of notifiable diseases reported through public health surveillance and the development of sanitation codes to the enforcement of environmental regulations (7).

The United States has a common law system, encompassing what frequently is referred to as "case law," in which judges interpret constitutions and statutes through written opinions that guide the application of the law. Within this system, the U.S. Supreme Court is the final authority over a hierarchy of courts. The hierarchy extends from municipal and other local courts that hear many public health cases, through the district, appeals, and supreme courts of each state, and finally, to federal district and appellate courts. Within this hierarchy, the opinions of an appeals court are binding on subordinate courts. Although state courts in one state do not bind the courts in other states, state courts often are influenced by courts in other states that have considered similar problems. This common law system allows judges to modify constitutions and statutes to adjust to changing conditions and unanticipated problems. Many cases with important ramifications for public health, such as the U.S. Supreme Court’s landmark decision *Jacobson v Massachusetts*, illustrate this process of applying constitutional provisions to public health situations that were not anticipated by the Constitution (8). Well-known areas of common law include contract, criminal, real property, and tort law, some of which have been important in public health (9). Tort law, for example, has addressed injuries caused by unsafe conditions. Although historically the body of judge-made tort law was almost entirely a common-law creation of judges, most states have now clarified and limited these judicial decisions by statute and regulation.

### Legal mechanisms, remedies, and tools

Under the sources of U.S. law described above, public health departments and other governmental agencies, as well as non-governmental organizations and parties, can opt to employ a variety of legal mechanisms, remedies, and tools for applying law to the prevention of diseases and injuries. Legal mechanisms represent several categories of governmental methods and interventions including not only the powers to tax and spend, but also the direct regulation of individuals (e.g., seatbelt requirements) and of businesses (e.g., licensure, inspections, fines, occupational safety standards) (3,7). Public health agencies also can turn to a broad set of remedies and sanctions to enforce regulations. Such remedies include civil sanctions — fines, suspension or revocation of licensure, and injunctions (also known as court orders) requiring termination of a defined activity required by law — and, in some instances, even criminal sanctions (7). Claims for damages under tort and property theories represent an additional legal tool for states, localities, individuals, or groups addressing public health problems. This tool has been used to protect the public from injury risks associated with products such as motor vehicles and tobacco (10).

### Jurisdictional levels

The jurisdictions at which mechanisms, remedies, and tools may be applied to public health problems span the federal, state, and local levels. While correspondence between the levels of enactment and application of laws can be straightforward, the fit and interplay of laws and mechanisms can be complicated for a multitude of public health policies and problems. For some problems, there may be a clear relationship between the source(s) of law and its jurisdictional application. For an ordinance enacted by a county commission to ban smoking in restaurants or entertainment venues, for example, the law will be highly specific to a narrowly defined geographic and political jurisdiction. For other problems, however, the relationship between the source(s) of law and the target public health problem may be extremely complex, involving a combination of federal, state, and local laws, and possibly even invoking the principle of preemption — the legal effect resulting when a superior governmental unit blocks an inferior governmental unit from regulation (7).

## Examples of legal frameworks in public health practice

Despite the foundational role of law in framing public health, as well as the important roles laws have played as interventions for public health problems, only a limited number of explicit, conceptual legal frameworks have been developed for preventing and controlling diseases and injuries. An historical example of the role of law in the modern public health movement is the Shattuck Report on sanitary conditions in Boston in 1850 (11). That report concluded with a proposed bill establishing a framework for public health regulation. This approach later was applied to zoning and city planning to improve the public’s health by separating residential housing from industrial areas, creating green spaces, and improving lighting and ventilation in multifamily housing.

A more recent example of the role of law in public health is a model — explicitly labeled as a legal framework — for improving the built environment (12). Other examples can be drawn from analyses of legal authorities related to public health problems such as acute disease and public health emergencies, environmental health, injuries, food-borne illnesses, and tobacco use-related diseases (13).

In outlining a model for modifying the design of the built environment to facilitate healthy behaviors and to create conditions for health, the authors noted that educating people about healthy lifestyles is by itself insufficient and that the built environment must allow for people to engage in healthy behaviors (12). The authors suggested that law be used as a tool to achieve the goals of a modified built environment and they proposed a framework of 5 legal approaches: 1) environmental regulation to reduce toxic emissions; 2) zoning ordinances to designate specific uses for areas; 3) building codes to set standards for structures; 4) taxation to encourage or discourage activities; and 5) spending to provide resources for projects that enhance the built environment (12). These legal approaches reflect not only constitutional principles but also the potential use of laws arising from a variety of federal, state, and local legislative enactments, and from administrative agencies.

While not explicit legal frameworks per se, some legal powers have been outlined as tools for addressing other public health problems. Legal authorities necessary for interventions during public health emergencies draw from a combination of constitutional sources, statutory enactments, and applications of the state’s police power — for example, the powers to seize property, abate nuisances, and implement personal control measures such as quarantine, isolation, and mandatory vaccination (14). To control and prevent food-borne diseases, public health and other government agencies at the federal, state, and local levels rely on a set of laws including federal statutes, such as the Federal Food, Drug, and Cosmetic Act and the Federal Meat Inspection Act, the uses of administrative law by agencies possessing delegated regulatory powers, such as the FDA and the Environmental Protection Agency (EPA), and myriad state and local legislated and delegated authorities (15). Other legal tool constructs exist for environmental health, tobacco control, and injury control (16,17). These constructs employ legislative and administrative laws and also identify a prominent role for litigation.

A final example is an analysis of laws related to potentially modifiable risk factors associated with coronary heart disease (CHD) (18). While this analysis was not advanced explicitly as a legal framework for the control and prevention of CHD, it nonetheless offers ideas and elements for a legal framework for addressing this and other chronic disease problems. The author of this analysis examined selected risk factors for CHD, such as smoking, in relation to laws most directly related to modifiable socio-environmental determinants for the factor (e.g., the Federal Cigarette Labeling and Advertising Act, state and local clean indoor air laws), as well as in relation to laws more remotely related to such determinants (e.g., state product liability laws, public health laws, and consumer fraud laws).

## Building the CDC’s public health law capacity in chronic disease prevention and control

Systematic legal frameworks in chronic disease prevention and control, like those described in the previous section, can be used to 1) assure that all potential legal avenues are considered; 2) provide a structure within which legal interventions can be monitored for appropriateness and effectiveness; and 3) assist in ensuring that laws, rules, orders, and regulations developed within these frameworks are implemented and enforced.

Specific legal frameworks could be derived for a number of issues within the arena of chronic disease prevention — prevention of heart disease, stroke, diabetes, asthma, obesity, cancer, or complications of diabetes, for example — and health promotion, such as reducing tobacco use, increasing physical activity, and improving nutrition. Indeed, the CDC’s National Center for Chronic Disease Prevention and Health Promotion (NCCDPHP) in collaboration with the CDC’s Public Health Law Program has launched the development of legal frameworks in 2 of these areas — prevention of cardiovascular disease and obesity — and aims to develop additional frameworks in other high-priority areas.

In addition to these issue-specific frameworks, an overarching legal framework could guide the development of legal tools for the entire range of chronic diseases, health behaviors, and environmental conditions. The framework could borrow effective legal tools from one area and apply them creatively to another. An overarching framework could be developed by incorporating the common elements of issue-specific frameworks and by analyzing cross-cutting issues in chronic disease prevention and health promotion.

To build the capacity of NCCDPHP to provide guidance and technical assistance in the emerging field of public health law, we plan to initiate several projects. First, a public health law work group has formed within NCCDPHP to oversee promising activities. An attorney-analyst and medical epidemiologist will coordinate efforts of a cross-NCCDPHP, multidisciplinary team with representatives from each category-specific division — for example, the Division of Adult and Community Health and the Office on Smoking and Health. Initial plans include a one-day meeting with external (non-CDC) program and public health law experts to develop the next steps in building capacity. Priorities include creating category-specific and overarching legal frameworks for chronic disease prevention and health promotion, hosting seminars on public health law and its current and potential uses in chronic disease, and expanding our ability to guide and collaborate with constituents in using legal tools for chronic disease prevention and health promotion.

## Existing resources

A growing body of information resources can assist public health professionals and others interested in building organizational capacity for using law as a tool for chronic disease prevention (17,19-24). The initial activities listed above and others will generate future articles in this and other journals. Readers may review statutes and case law within their own jurisdictions and also consult their legal counsel on laws relating to their program's goals. Finally, public health conferences increasingly are offering educational sessions and programs on laws for preventing chronic diseases. The CDC Public Health Law Program Web site offers information on 2 past conferences (25) and on the upcoming conference, *The Public's Health and the Law in the 21st Century*, to be held June 14–16, 2004, in Atlanta, Ga (26).

## Conclusions

This paper outlined the variety of legal tools, remedies, and mechanisms available to public health practitioners and policy makers for achieving public health goals and also examined law as a tool for expanding strategies for preventing and controlling chronic diseases. We emphasize that the use of law should complement, not supplant, existing strategies based on well-established principles of public health practice (27). Law can bolster existing strategies when used prudently by public health practitioners who have a clear understanding of how it shapes public health infrastructure and can promote program goals.

In addition to giving examples of the effectiveness of laws in public health, we described the broad jursiprudential landscape upon which legal frameworks address chronic diseases across a wide array of programs not limited to officially designated public health agencies. Medicare and Medicaid programs, for example, set policies that determine access for large segments of the U.S. population to screening and secondary prevention activities for a number of chronic disease risk factors and conditions. Similarly, the EPA and its state counterparts determine to a large extent exposure levels to airborne and waterborne toxins and to particulates linked to cancer, asthma, and other chronic diseases. Municipal water systems, many independent of officially designated public health agencies, influence oral health through fluoridation policies. Even federal and state revenue agencies, while established largely for other purposes, affect the public’s health through policies that assign taxable status to preventive medical treatments.

Legal frameworks provide exciting opportunities for expanding the spectrum of effective public health strategies. In collaboration with the CDC’s Public Health Law Program, other legal experts, and our external partners, NCCDPHP will continue to explore the development, dissemination, and use of these legal frameworks for the prevention and control of chronic diseases.
